# Fracture strength of conventional versus digital interim crowns after thermal cycling

**DOI:** 10.1186/s12903-026-09074-z

**Published:** 2026-07-01

**Authors:** Işıl Sarıkaya, Tuğçe Koç, İlknur Usta Kutlu

**Affiliations:** Department of Prosthodontics, Faculty of Dentistry, Gaziosmanpaşa University, Tokat, 60100 Turkey

**Keywords:** Computer-aided design, Three-dimensional printing, Temporary dental restoration, Dental prosthesis, Polymethyl methacrylate

## Abstract

**Background:**

Provisional restorations are essential components of fixed prosthodontic treatment. This study compared the fracture strength and failure modes of provisional crowns fabricated using conventional direct, conventional indirect, CAD/CAM 3D printing, and CAD/CAM milling techniques after thermal aging.

**Methods:**

A total of 48 provisional crowns were fabricated across four groups (*n* = 12 per group): conventional direct technique using dual-cured composite resin (Tempsmart DC, GC Corporation, Tokyo, Japan), conventional indirect technique using autopolymerized PMMA (Temdent Classic, Schütz Dental GmbH, Rosbach, Germany), CAD/CAM 3D printing using light-cured resin (ResMach Interim, ResMach, Istanbul, Turkey), and CAD/CAM milling using pre-polymerized PMMA (Acryx, Akrodent, Istanbul, Turkey). All crowns were cemented onto standardized cobalt-chromium dies and subjected to 10,000 thermocycles alternating between 5 °C and 55 °C (30-second dwell time; 5-second transfer time). Fracture strength testing was performed using a universal testing machine at a crosshead speed of 1 mm/min until failure, with maximum loads recorded in Newtons. Fracture modes were classified using Burke’s classification through visual and photographic analysis. Statistical analysis was performed using one-way ANOVA and Tamhane’s T2 post-hoc test (*p* < 0.05).

**Results:**

The highest fracture strength was recorded in the Acryx group (1511.67 ± 140.73 N), significantly higher than all other groups (*p* < 0.001). The ResMach Interim group showed the lowest fracture strength (581.62 ± 51.86 N). Conventional groups (Tempsmart DC: 716.58 ± 104.81 N; Temdent Classic: 792.85 ± 83.70 N) had intermediate values. One-way ANOVA revealed a large effect of fabrication technique on fracture strength (η² = 0.857). Acryx exhibited predominantly Class II fractures (75.0%), while other groups showed Class I fractures (83.3–100%).

**Conclusions:**

CAD/CAM-milled PMMA crowns demonstrated superior fracture resistance and more favorable ductile fracture patterns compared to conventional and 3D-printed crowns after thermal aging, suggesting these materials may be preferred for long-term provisional use in high-stress clinical scenarios such as posterior region applications.

## Background

Provisional restorations are a fundamental component of fixed prosthodontic treatment, serving as temporary restorations during the period between tooth preparation and placement of the definitive prosthesis [1, 2]. These restorations protect prepared tooth structures and pulp tissues, maintain occlusal relationships and periodontal health, and provide acceptable esthetics and function throughout treatment [1, 3, 4]. In addition, provisional restorations serve as diagnostic tools that allow evaluation of functional and esthetic outcomes prior to final restoration delivery [1, 3–5].

Material selection is a major determinant of the clinical performance and longevity of provisional restorations [6]. Polymethyl methacrylate (PMMA) resins have been widely used for decades due to their cost efficiency and ease of manipulation [1, 3, 7]. However, conventional PMMA materials exhibit several limitations, including polymerization shrinkage, exothermic heat release during setting, limited wear resistance, and potential pulpal irritation caused by residual monomer [8, 9]. Bis-acrylic composite resins were subsequently introduced to overcome these disadvantages, offering reduced shrinkage, improved mechanical properties, enhanced marginal adaptation, and lower exothermic reactions [3, 8, 10]. Despite these improvements, findings regarding fracture resistance remain inconsistent, with some studies reporting superior performance of bis-acrylic materials [11–14], while others suggest that fabrication techniques and material-specific properties may have a greater influence than material type alone [15].

The mechanical durability of provisional restorations is essential for maintaining clinical function and patient comfort. These restorations are subjected to complex oral conditions, including cyclic masticatory forces, thermal fluctuations associated with food and beverage intake, and continuous chemical exposure within the oral environment [16, 17]. Among mechanical properties, fracture resistance is considered a key determinant of clinical longevity [16, 17]. Restoration failure may be influenced by parafunctional habits such as bruxism, occlusal discrepancies, insufficient occlusal clearance, and the structural demands of long-span fixed partial dentures [4, 18, 19].

The integration of computer-aided design and computer-aided manufacturing (CAD/CAM) technologies has significantly advanced the fabrication of provisional restorations. CAD/CAM systems employ both subtractive manufacturing through milling and additive manufacturing via three-dimensional (3D) printing [8, 10, 20, 21]. These digital workflows offer improved dimensional accuracy, enhanced reproducibility, reduced chairside fabrication time, and the potential for improved mechanical performance due to industrial material processing [20, 21]. CAD/CAM-milled PMMA materials produced under controlled industrial conditions demonstrate greater material homogeneity, minimal internal porosity, and reduced residual monomer content. Several studies have reported higher fracture resistance for milled PMMA compared with conventionally fabricated provisional materials [10, 12, 20]; however, conflicting findings persist, with some investigations reporting comparable or lower fracture resistance relative to bis-acrylic resins under specific conditions [22].

Thermal aging is a critical factor in evaluating the performance of provisional restorations, as temperature fluctuations and prolonged exposure to moisture may induce dimensional changes and degradation of mechanical properties [23, 24]. Recent studies indicate that thermal aging affects provisional materials and fabrication techniques to varying degrees [23, 24]. Comparative evidence remains limited across contemporary fabrication methods, including bis-acrylic composite resins, autopolymerized PMMA, CAD/CAM-milled materials, and additive manufacturing. Additive manufacturing, particularly the 3D printing of photopolymerizable dental resins, has emerged as a promising fabrication approach. Despite advantages such as design flexibility and fabrication efficiency, evidence regarding the mechanical performance of provisional restorations fabricated through different techniques remains limited and inconsistent [8, 17, 24–26]. Factors such as material composition, fabrication method, and technique-specific variables—including build orientation, interlayer bonding strength, layer thickness, and post-curing protocols in 3D printing—may substantially influence fracture behavior [27, 28].

Despite the expanding range of available provisional restorative materials and fabrication technologies, fractures remain among the most frequent clinical complications, contributing to additional chairside time, patient discomfort during replacement procedures, and increased treatment costs. Although a number of comparative investigations have been conducted, direct head-to-head comparisons among bis-acrylic composites, autopolymerized PMMA, CAD/CAM-milled pre-polymerized PMMA, and 3D-printed light-cured resins under standardized thermocycling conditions remain limited [7, 17]. Furthermore, failure mode characterization—specifically the clinical distinction between ductile and brittle fracture behavior and its implications for repairability and patient safety—has received insufficient attention in the existing literature. To address these gaps, the objective of this in vitro study was to evaluate and compare the fracture resistance and failure modes of provisional crowns fabricated using four different techniques—conventional direct (bis-acrylic composite), conventional indirect (autopolymerized PMMA), CAD/CAM 3D printing (light-cured resin), and CAD/CAM milling (pre-polymerized PMMA)—following standardized thermal aging. The null hypothesis was that fabrication technique would not significantly influence fracture resistance or failure patterns after thermal aging.

## Methods

### Sample size calculation

Sample size determination was performed using G*Power software (Version 3.1.9.7; Heinrich Heine Universität Düsseldorf, Germany). Based on previously reported mean fracture strength data (Abdullah et al., 2016) [29], in which values ranged from 361.01 ± 21.61 N to 802.23 ± 111.29 N across fabrication groups, the between-group differences were substantially larger than within-group variability, yielding an effect size of f = 1.7 even when the largest group standard deviation was used as a conservative pooled estimate. The calculation employed an alpha error probability of 0.05 and statistical power of 90%, indicating a minimum requirement of 12 specimens per group and a total of 48 provisional crowns distributed across four groups (*n* = 12 per group).

### Master die fabrication

**Fig. 1:**
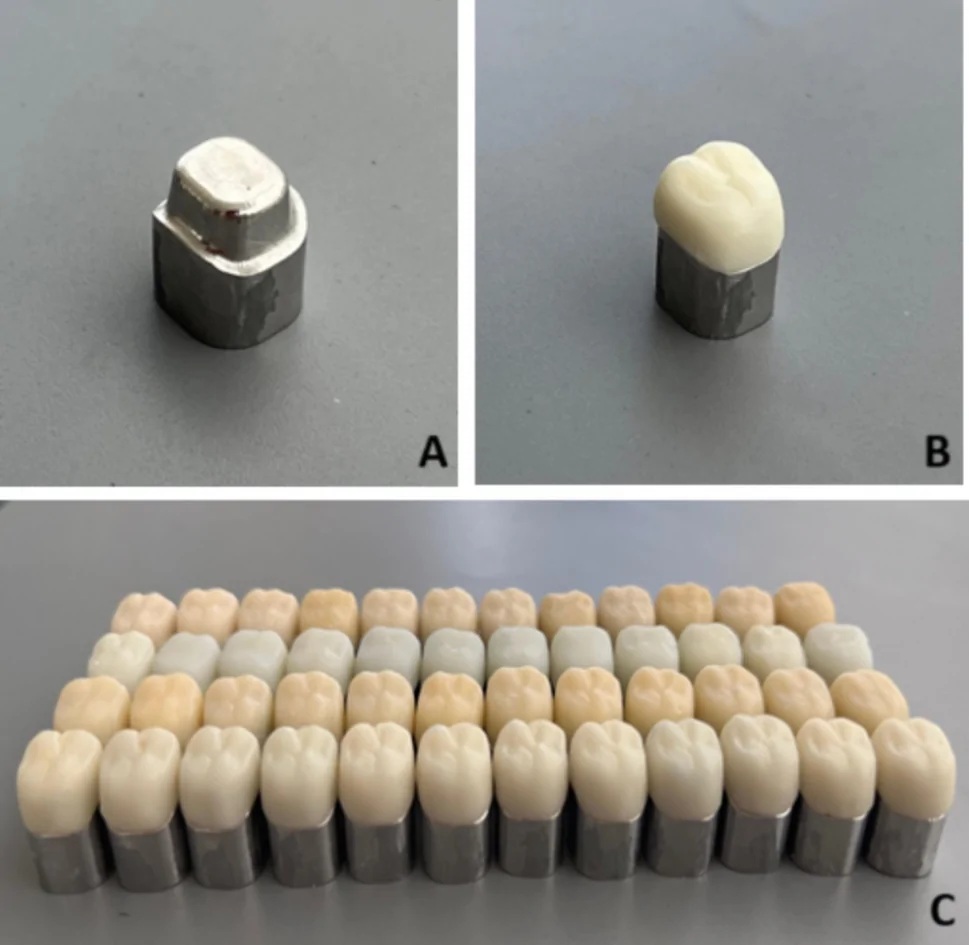
Preparation of Provisional Crown Specimens. **A** Metal master model fabricated for crown production. **B** Provisional crown positioned on the metal die. **C** All fabricated specimens arranged and ready for testing

A standardized cobalt-chromium alloy master die model was digitally designed using SolidWorks software (SolidWorks Corp., Waltham, MA, USA), replicating a prepared maxillary first molar appropriate for full-coverage crown restoration. The preparation design incorporated 2 mm of occlusal reduction, a 1 mm chamfer finish margin, and 12 degrees of total occlusal convergence [1]. Using this digital design as a template, 48 identical Co-Cr die models (CC Solar; Camcube) were fabricated via CAD/CAM milling technology (M30; Camcube). The CAD/CAM milling process ensured dimensional accuracy and consistency across all specimens, with each die precisely replicating the standardized tooth preparation geometry established in the digital design. Material details for all fabrication groups are provided in Table 1, and representative specimens are shown in Fig. 1.

**Table 1:** Materials used in the study groups

Material (*n* = 12)	Main components	Manufacturer	Manufacturing method
Tempsmart DC	Dual-cured composite resin	GC Corporation, Tokyo, Japan	Conventional direct
Temdent Classic	Cold-curing PMMA	Schütz Dental GmbH, Rosbach, Germany	Conventional indirect
ResMach Interim	UV-curing resin	ResMach, Istanbul, Turkey	CAD/CAM 3D printing
Acryx	PMMA block	Akrodent, Istanbul, Turkey	CAD/CAM milling

*CAD/CAM* computer-aided design/computer-aided manufacturing, *PMMA* polymethylmethacrylate

### Digital workflow and crown design

One of the fabricated master dies was digitized using an intraoral scanner (Trios 3; 3Shape A/S, Copenhagen, Denmark) to create a digital reference model. Crown design was performed using CAD software (Exocad GmbH, Darmstadt, Germany) with standardized parameters: 0.5 mm coping thickness [30, 31] and 50 μm cement space beginning 0.5 mm occlusal to the finish line [7]. The finalized designs were exported as standard tessellation language (STL) files for subsequent fabrication processes across all digital manufacturing groups.

#### Group 1: Conventional direct (Tempsmart DC)

An initial full-coverage provisional crown was fabricated via CAD/CAM milling to serve as an anatomical template for the conventional groups. A two-stage silicone index (C-Silicone; Zhermack SpA, Badia Polesine, Italy) was created from this template crown to enable consistent reproduction of crown morphology across all conventional specimens. For the direct technique, dual-cured composite resin (Tempsmart DC; GC Corporation, Tokyo, Japan) was dispensed directly into the silicone matrix according to manufacturer specifications, seated onto the master die under constant pressure, and allowed to polymerize completely. Excess material was removed and specimens were finished and polished following standard laboratory protocols.

#### Group 2: Conventional indirect (Temdent Classic)

Using the identical silicone index prepared for Group 1, autopolymerized PMMA (Temdent Classic; Schütz Dental GmbH, Rosbach, Germany) was mixed according to manufacturer-recommended powder-to-liquid ratios. The material was applied to the silicone matrix, the die was placed in the matrix and left to polymerize under controlled laboratory conditions. Following complete polymerization, specimens underwent finishing and polishing procedures identical to those employed for Group 1.

#### Group 3: CAD/CAM 3D printing (ResMach Interim)

The previously saved STL files were employed to fabricate provisional crowns using additive manufacturing technology. A light-cured resin material (ResMach Interim; ResMach, Istanbul, Turkey) was processed using a stereolithography (SLA) 3D printer (Anycubic Photon Mono M7; Anycubic, Shenzhen, China). All crowns were fabricated with a layer thickness of 100 μm in a horizontal (0°) build orientation relative to the build platform, with support structures positioned on the occlusal surface in accordance with the manufacturer’s recommendations. These parameters are consistent with established protocols in the literature for 3D-printed provisional restorations [17, 25, 26]. Upon completion of printing, support structures were carefully removed under magnification. Printed specimens were subsequently cleaned in isopropyl alcohol for 10 min to eliminate residual uncured resin, followed by post-curing in a dedicated light-curing unit (Anycubic Wash and Cure Machine; Anycubic, Shenzhen, China) for 15 min to ensure complete polymerization and optimal mechanical properties [27]. All specimens were finished and polished to achieve surface quality comparable to that of the other experimental groups.

#### Group 4: CAD/CAM milling (Acryx)

Pre-polymerized PMMA blocks (Acryx; Akrodent, Istanbul, Turkey) were milled using a milling machine (Redon Hybrid; Redon, Istanbul, Turkey) based on the stored STL files. The milled provisional crowns required no additional sintering or post-processing beyond standard finishing and polishing procedures, as the blocks were already fully polymerized during industrial manufacturing.

### Specimen cementation

All provisional crowns were cemented onto their respective cobalt-chromium dies using zinc oxide eugenol-based temporary luting cement (Cavex Temporary Cement; Cavex Holland BV, Haarlem, Netherlands) [32]. A standardized cementation protocol was implemented to ensure consistency: each crown was initially seated with finger pressure for 10 s by the same investigator to ensure correct positioning, followed by application of a static load of 2 kg for 5 min [32]. This protocol ensured uniform cement film thickness and eliminated variations in seating force across all specimens.

### Thermocycling procedure

To replicate intraoral thermal conditions encountered during normal function, all cemented specimens were subjected to thermocycling in accordance with ISO TR 11,405 standards. The thermocycling regimen consisted of 10,000 cycles alternating between water baths maintained at 5 °C and 55 °C (Thermocycler SD Mechatronic; Kulzer, Hanau, Germany). Each cycle included 30-second dwell times at each temperature extreme with 5-second transfer intervals between baths. This standardized protocol simulates approximately one year of clinical service, inducing both thermal stress and potential hydrolytic degradation of the provisional materials [23].

### Fracture strength testing

**Fig. 2:**
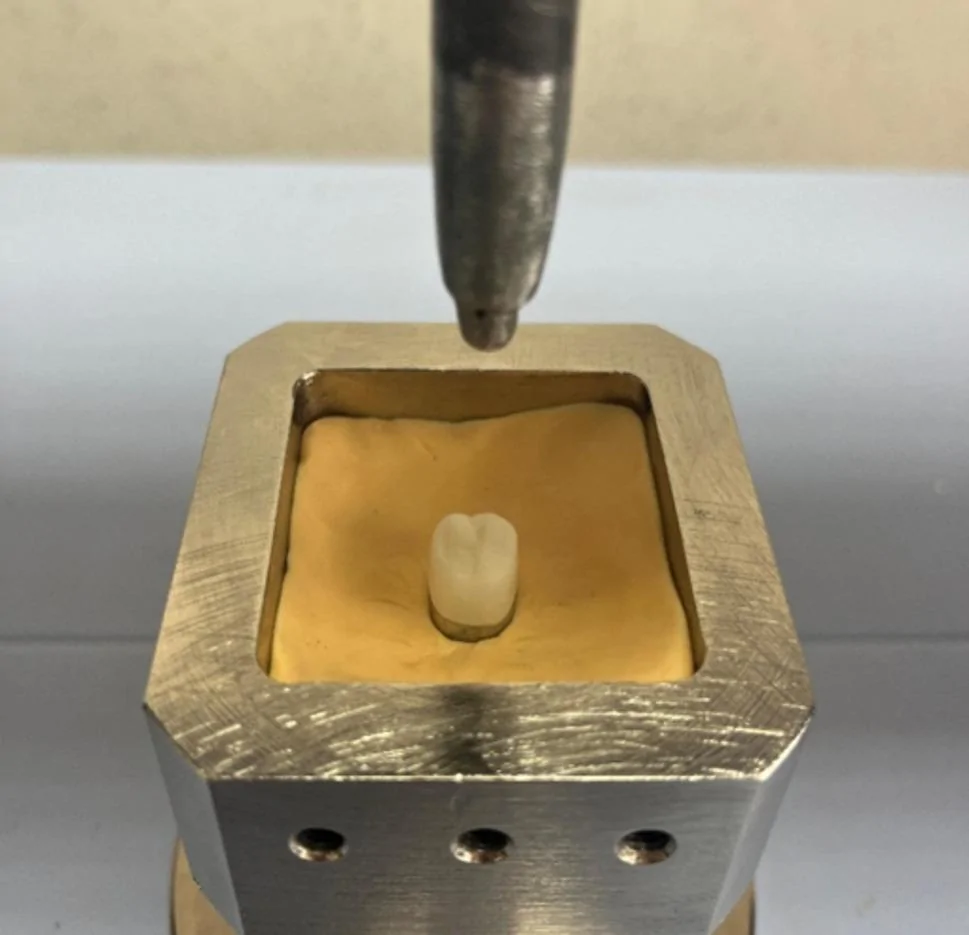
Specimen mounted on the universal testing machine. The hemispherical stainless-steel indenter (6 mm diameter) is positioned at the central fossa with PTFE tape interposed for uniform load distribution

Mechanical testing was conducted using a universal testing machine (AGS-X; Shimadzu Corporation, Kyoto, Japan). Each thermocycled specimen was positioned in a custom-fabricated holding fixture designed to ensure proper axial alignment during testing. A compressive load was applied perpendicular to the occlusal surface at the central fossa using a hemispherical stainless-steel indenter (diameter 6 mm) at a crosshead speed of 1 mm/min until catastrophic failure occurred. To compensate for minor surface irregularities and ensure uniform load distribution across the occlusal surface, a thin polytetrafluoroethylene (PTFE/Teflon) tape was interposed between the indenter and the crown. The maximum load at failure was recorded in Newtons. The testing setup is shown in Fig. 2.

### Failure mode analysis

Following fracture strength testing, all specimens were systematically examined to determine failure patterns and classify fracture modes. Each fractured crown was evaluated visually under magnification and documented photographically for permanent record. Two independent examiners, calibrated prior to assessment and blinded to group allocation, categorized each specimen according to Burke’s classification system [33] (Table 2).

**Table 2:** Classification of the failure modes according to Burke [33]

Classification	Pattern of fracture
Class I	Minimal fracture or crack in crown
Class II	Less than half of crown lost
Class III	Crown fracture through midline; half of crown displaced or lost
Class IV	More than half of crown lost
Class V	Severe fracture of tooth and/or crown

### Statistical analysis

Statistical analysis was performed using SPSS software (Version 22.0; IBM Corp., Armonk, NY, USA). Descriptive statistics including means, standard deviations, minimum and maximum values, and 95% confidence intervals were calculated for fracture strength values in each group. The assumption of normality was assessed using the Shapiro-Wilk test and homogeneity of variances was evaluated using Levene’s test. Due to heterogeneity of variances among groups, one-way analysis of variance (ANOVA) was employed to detect differences in fracture strength among the four fabrication techniques, followed by Tamhane’s T2 post-hoc test for pairwise comparisons. In addition to *p*-values, effect sizes were calculated using Partial Eta Squared to evaluate the magnitude of the differences between the groups. The values were interpreted as small (0.01), medium (0.06), or large (0.14). Statistical significance was established at *p* < 0.05 for all analyses.

## Results

The fracture strength data obtained from all four experimental groups demonstrated distinct performance profiles following thermal aging and mechanical testing. Mean fracture strength values, standard deviations, minimum and maximum values, 95% confidence intervals, and effect size are presented in Table 3.

**Table 3:** Fracture strength (*N*) and effect size comparison of the groups

Material	Mean (*N*)	SD	Min	Max	95% CI Lower	95% CI Upper	η²	*p*	Post-hoc comparisons
Tempsmart DC (1)	716.58	104.81	522.80	823.40	649.99	783.17	0.857	< 0.001*	1–3 (*p* = 0.006) 1–4 (*p* < 0.001) 2–3 (*p* < 0.001) 2–4 (*p* < 0.001) 3–4 (*p* < 0.001)
Temdent Classic (2)	792.85	83.70	646.50	922.00	739.67	846.03	–	–	–
ResMach Interim (3)	581.62	51.86	479.80	649.40	548.67	614.57	–	–	–
Acryx (4)	1511.67	140.73	1223.00	1700.50	1422.25	1601.08	–	–	–

(1) Tempsmart DC; (2) Temdent Classic; (3) ResMach Interim; (4) Acryx

Post-hoc comparisons listed indicate pairs with statistically significant differences (Tamhane's T2 test)

*SD* standard deviation, *CI* confidence interval, *η*² eta-squared (effect size for one-way ANOVA; Cohen's classification: > 0.14 = large)

– not applicable

Among the four fabrication methods evaluated, the Acryx group (CAD/CAM milling) exhibited the highest mean fracture strength at 1511.67 ± 140.73 N, followed by Temdent Classic (conventional indirect) at 792.85 ± 83.70 N, Tempsmart DC (conventional direct) at 716.58 ± 104.81 N, and ResMach Interim (CAD/CAM 3D printing) at 581.62 ± 51.86 N (Table 3).

**Fig. 3:**
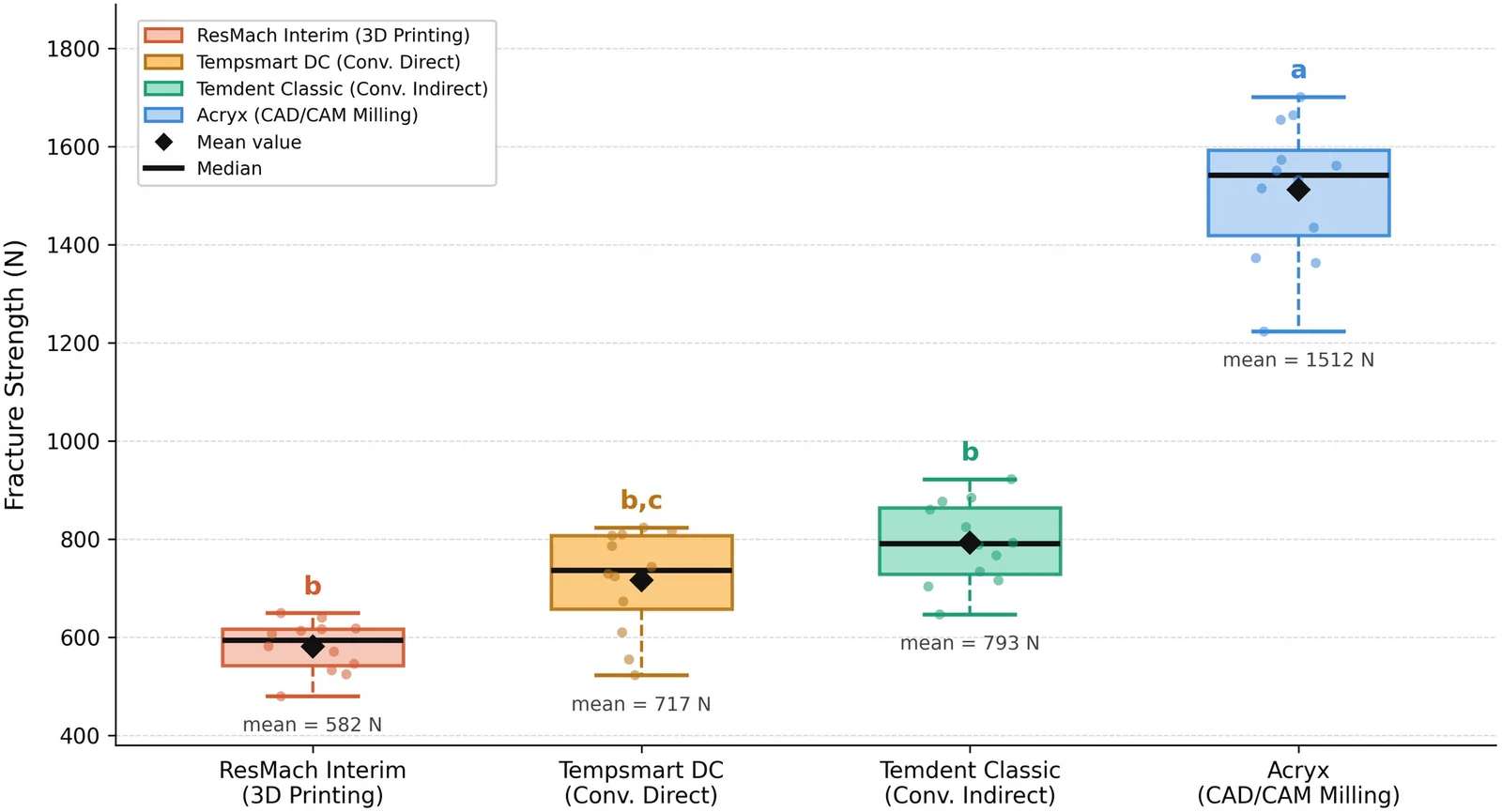
Distribution of fracture strength values (*N*) of provisional crowns by fabrication group. Boxes represent the interquartile range (Q1–Q3); horizontal lines within boxes indicate median values; diamond symbols (◆) indicate group means; whiskers extend to minimum and maximum observed values; individual data points (*n* = 12 per group) are shown as jittered dots. Different letters indicate statistically significant differences between groups (*p* < 0.05, Tamhane T2 post-hoc test)

Statistical analysis using one-way ANOVA revealed significant differences in fracture strength among the four groups (*p* < 0.001). The eta-squared value was η² = 0.857, indicating that fabrication technique accounted for approximately 85.7% of the total variance in fracture strength, corresponding to a large effect size according to Cohen’s classification. Post-hoc pairwise comparisons using Tamhane’s T2 test demonstrated that the Acryx group significantly outperformed all other groups (*p* < 0.001 for all comparisons). The ResMach Interim group showed significantly lower fracture strength compared to both conventional groups and the Acryx group (*p* < 0.001). No statistically significant difference was observed between the two conventional groups, Tempsmart DC and Temdent Classic (*p* > 0.05). The distribution of fracture strength values by group is illustrated in Fig. 3.

**Fig. 4:**
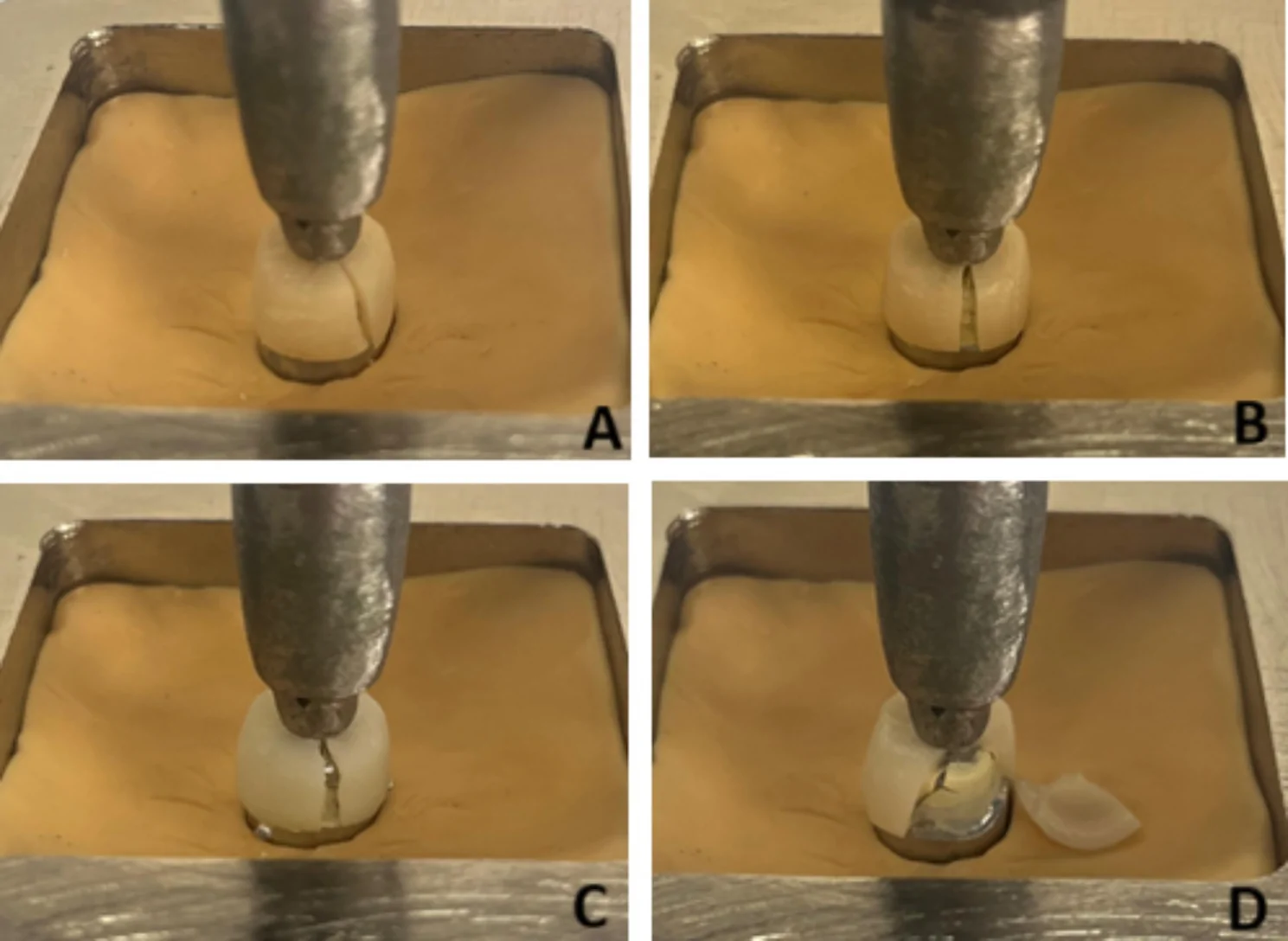
Fracture patterns of provisional crowns according to Burke’s classification distributed across the four fabrication groups

Regarding failure mode analysis, distinct fracture pattern distributions were observed among the groups. The Acryx group exhibited predominantly Class II fractures (75.0% of specimens), characteristic of ductile failure behavior. In contrast, the other three groups demonstrated primarily Class I fractures, with Tempsmart DC showing 83.3%, Temdent Classic showing 91.7%, and ResMach Interim showing 100% Class I fractures, all indicative of brittle failure patterns. No Class III, IV, or V fractures were observed in any experimental group. The distribution of fracture patterns is presented in Fig. 4.

## Discussion

This investigation systematically assessed the fracture resistance and failure characteristics of provisional crowns fabricated through four distinct methodologies following simulated clinical aging. Statistical analysis led to rejection of the null hypothesis, as significant differences in maximum fracture forces were detected among groups (*p* < 0.001), with a large effect size (η² = 0.857) confirming the practical significance of these differences. However, the two conventional fabrication methods (direct and indirect) demonstrated statistically comparable performance (*p* > 0.05).

The CAD/CAM-milled PMMA specimens (Acryx) demonstrated the highest fracture strength at 1511.67 ± 140.73 N, substantially exceeding typical posterior masticatory forces reported in the literature (500–900 N) [7]. This superior mechanical performance can be primarily attributed to the industrial polymerization process employed in pre-polymerized block manufacturing, which yields highly homogeneous material structures characterized by minimal internal porosity and substantially reduced residual monomer content [10, 29].

These observations align consistently with investigations documenting enhanced mechanical characteristics of milled PMMA relative to both conventional and 3D-printed alternatives [7, 8, 24, 28, 29]. Abdullah and colleagues reported that CAD/CAM-milled provisional crowns achieved fracture strength values of 1289 ± 56 N, surpassing conventional PMMA due to diminished water sorption characteristics and elevated surface hardness [29]. The uniform microstructure characteristic of industrially pre-polymerized PMMA blocks substantially reduces internal defect formation and stress concentration sites, thereby contributing directly to enhanced mechanical performance [20]. Contemporary systematic reviews have further validated the enhanced marginal and internal adaptation of CAD/CAM-fabricated provisional restorations, which may facilitate improved load distribution patterns and enhanced mechanical stability during clinical function [34].

The 3D-printed specimens (ResMach Interim) recorded the lowest fracture strength at 581.62 ± 51.86 N, a finding likely attributable to their layer-by-layer construction methodology, potential incomplete post-polymerization between layers, and inherent susceptibility to hydrolytic degradation [17, 24–26]. The anisotropic nature of additively manufactured materials results in comparatively weaker interlayer bonding relative to the homogeneous three-dimensional structure characteristic of milled materials [25, 26]. Post-curing protocols exert substantial influence on the final mechanical properties of 3D-printed resins, with inadequate post-curing resulting in incomplete polymerization reactions and diminished cross-link density within the polymer matrix [27]. Aati and colleagues demonstrated that prolonged post-curing light exposure significantly enhanced the physico-mechanical properties of 3D-printed dental materials [27]. Similarly, previous investigations have reported substantial variability in mechanical performance among 3D-printed provisional restorations, influenced predominantly by resin composition, build orientation, layer thickness, and post-curing protocol selection, thereby emphasizing the critical necessity for optimized printing parameters [25, 26, 28].

Several recent investigations have reported comparable mechanical performance between optimized 3D-printed resins and bis-acrylic materials [7, 24], suggesting that ongoing advances in resin chemistry and printing technology may progressively narrow this performance differential in future material generations. Specific optimization strategies including layer thickness reduction to below 50 μm, strategic build orientation selection, and implementation of enhanced post-curing protocols could substantially improve the mechanical performance of 3D-printed restorations [24–26, 28]. The continued development of novel 3D-printable resins incorporating enhanced mechanical properties and reduced anisotropic behavior represents a particularly promising avenue for future investigation [35].

The conventional groups (Tempsmart DC and Temdent Classic) demonstrated intermediate fracture strength values, potentially limited by inherent factors associated with manual fabrication including mixing artifacts, inadvertent porosity introduction, and incomplete polymerization reactions [34]. No statistically significant difference was detected between the conventional direct (Tempsmart DC) and conventional indirect (Temdent Classic) groups, a finding that aligns with previous comparative studies evaluating bis-acrylic and autopolymerized PMMA materials under similar testing conditions [22]. Digholkar and colleagues observed that conventional PMMA formulations exhibited reduced flexural strength compared to CAD/CAM-milled materials, thereby reinforcing the limitations associated with manual fabrication techniques [36]. The inadvertent incorporation of voids and irregularities during manual production procedures creates stress concentration points within the material matrix that compromise overall mechanical performance [10].

Fracture pattern analysis revealed distinct ductile failure behavior in Acryx specimens (75.0% demonstrating Class II fractures) contrasting markedly with the predominantly brittle failure observed in other groups (Class I fractures: 83.3–100%), suggesting that milled PMMA may offer greater clinical suitability for high-stress applications; however, in vivo validation is required before definitive recommendations can be made [7, 34]. The implemented thermocycling protocol (10000 cycles) is widely accepted as representing one year of typical clinical service, inducing both hydrolytic degradation and cumulative thermal stress within the materials [23]. Furthermore, the repairability and surface treatment compatibility of CAD/CAM provisional materials represent additional clinical considerations that may influence long-term performance [30, 37]. Additionally, the choice of cement type may influence fracture resistance outcomes through its effect on stress distribution at the crown-die interface; however, as all specimens were cemented under identical conditions using zinc oxide eugenol-based cement, the relative comparison between fabrication groups is considered reasonable. Zinc oxide eugenol-based cement was selected for its widespread clinical use as a temporary luting agent, its well-documented biocompatibility and compatibility with provisional resin materials, and its ease of removal without adversely affecting the mechanical behavior of the restorations [10, 32]. While static fracture strength tests provide valuable data regarding the ultimate load-bearing capacity of interim restorative materials, in the oral environment, restorations are subjected to repetitive cyclic masticatory forces as well as thermal stresses, which may lead to clinical failure at loads significantly lower than the static fracture peak due to fatigue. Although thermal aging was performed in the present study to simulate the oral environment, the lack of dynamic mechanical loading remains a limitation.

Several study limitations warrant acknowledgment. The in vitro experimental design, while enabling precise control of testing variables, cannot fully replicate the complex conditions present in the clinical oral environment. The application of static loading until catastrophic failure, while standard in material testing protocols, does not precisely simulate the cyclic fatigue loading patterns experienced during typical clinical function; accordingly, the absence of cyclic fatigue simulation should be considered when extrapolating these findings to clinical settings, as intraoral failures may occur at lower force thresholds due to cumulative fatigue mechanisms [17]. In this study, Co-Cr dies were used to ensure a high degree of standardization. However, the high elastic modulus of Co-Cr does not fully replicate the mechanical behavior of natural dentin. This rigidity may influence stress distribution and potentially lead to higher fracture strength values compared to clinical scenarios. Future research utilizing substrates with dentin-like elasticity could provide further insights into the clinical performance of these restorations. The failure mode analysis in this study was limited to visual inspection. While this method is practical for identifying primary failure patterns, it lacks the detailed topographical data provided by microscopic evaluation, such as Scanning Electron Microscopy (SEM). Consequently, the nuances of crack propagation and surface characteristics were not fully explored, which remains a limitation of the present study. The study evaluated a single tooth type (maxillary first molar) and employed a single representative material for each fabrication technique, potentially limiting the generalizability of findings. Future investigations should incorporate in vivo validation studies, dynamic cyclic fatigue testing protocols, evaluation of diverse tooth preparations including anterior teeth and multi-unit prostheses, systematic optimization of 3D printing parameters, and longitudinal clinical performance assessments to validate in vitro findings [17, 24–26, 28, 35].

## Conclusions

CAD/CAM-milled PMMA crowns (Acryx) exhibited superior fracture resistance and favorable ductile failure patterns following thermal aging, suggesting they may be preferred for long-term provisional use in high-stress scenarios such as posterior region applications. Clinical extrapolation should be approached with caution given the inherent limitations of in vitro static loading designs. 3D-printed crowns demonstrated the lowest fracture strength among evaluated groups, indicating a clear need for continued advancements in material formulations and post-processing protocols. Conventional fabrication techniques demonstrated adequate performance for short-term, moderate-stress applications. Clinical material selection should be carefully tailored to individual patient needs and anticipated stress conditions, with CAD/CAM-milled PMMA representing a favorable option for long-term provisional restorations or high-stress clinical cases, pending further in vivo confirmation.

## Data Availability

The datasets generated and/or analysed during the current study are available from the corresponding author on reasonable request.
